# Preparation and *in vivo* evaluation of a highly skin- and nail-permeable efinaconazole topical formulation for enhanced treatment of onychomycosis

**DOI:** 10.1080/10717544.2019.1687612

**Published:** 2019-11-18

**Authors:** Byung Chul Lee, Rudra Pangeni, Jungtae Na, Kyo-Tan Koo, Jin Woo Park

**Affiliations:** a Department of Dermatology, College of Medicine, Chung-Ang University, Seoul, Republic of Korea;; b Department of Medicine, Graduate School, Chung-Ang University, Seoul, Republic of Korea;; c College of Pharmacy and Natural Medicine Research Institute, Mokpo National University, Jeonnam, Republic of Korea;; d BioBelief Co., Ltd., Seoul, Republic of Korea

**Keywords:** Efinaconazole, topical solution, onychomycosis, skin permeability, nail infiltration, antifungal activity

## Abstract

Onychomycosis is a progressive fungal infection of the nails that involves the deeper nail layer and nail bed. It is important to maintain sufficient drug concentration in the diseased tissues after topical application. In this study, a stable topical delivery system for efinaconazole (EFN) was designed to enhance absorption potential through the skin and nail plate by incorporating ethanol, diethylene glycol monoethyl ether (Transcutol P) and isopropyl myristate, and cyclomethicone into the topical solution as a delivery vehicle, permeation enhancers, and a wetting agent, respectively. In addition, the stability of EFN in the formulation was significantly improved by adding butylated hydroxytoluene, diethylenetriamine pentaacetic acid, and citric acid as an antioxidant, chelating agent, and pH-adjusting agent, respectively, without discoloration. The optimum EFN formulation (EFN-K) showed 1.46-fold greater human skin permeation than that of the reference control (commercial 10% EFN topical solution). Furthermore, after a 24-hour incubation, the amount of infiltrated EFN from EFN-K in the human nail plate was 4.11-fold greater than that of the reference control, resulting in an 89.7% increase in nail flux at 7 days after treatment. EFN-K significantly accelerated structural recovery of the keratin layer in a *Trichophyton mentagrophytes*-infected guinea pig onychomycosis model, decreasing the mean viable fungal cell count by 54.3% compared to the vehicle-treated group after once-daily treatment for 4 weeks. Thus, the accelerated skin and nail penetration effect of EFN-K is expected to achieve good patient compliance, and improve the complete cure rate of onychomycosis.

## 1. Introduction

Onychomycosis is one of the most chronic and prevalent nail disorders, and is commonly caused by fungi, such as dermatophytes (*Trichophyton rubrum* and *Trichophyton mentagrophytes*), non-dermatophyte molds, and yeasts (*Candida albicans*), which more frequently affect toenails than fingernails (Shirwaikar et al., 2008; Lasenna & Tosti, 2015). Onychomycosis is a major public health concern due to the typically poor response to therapy and associated significant clinical, physical, and psychological issues (Solis-Arias & Garcia-Romero, 2017; Gupta & Mays, 2018). Moreover, the treatment of onychomycosis should only be performed after clinical assessment and microbiological confirmation, as some patients with multiple diseases, such as diabetes and onychomycosis, are at greater risk of developing complications. In addition, due to the low penetration of drugs through the nail plate, high recurrence rate, low clinical efficacy, and patient preference for topical solutions, the need for an efficacious treatment for onychomycosis remains unmet (Singal & Khanna, 2011; Lasenna & Tosti, 2015). Furthermore, oral antifungals currently used in the treatment of onychomycosis have several limitations, including in terms of safety due to hepatotoxicity and drug–drug interactions (Lasenna & Tosti, 2015; Matsuda et al., 2016). Moreover, due to the limited vascular access to the nail bed and its barrier properties, systemic treatment of nail diseases is ineffective (Kushwaha et al., 2017). Recent studies also suggested roles for azoles in reproductive and developmental toxicity in both humans and laboratory animals, so long-term systemic exposure is not recommended (Lee et al., 1992; Keber et al., 2011; Glynn et al., 2015). On the other hand, topical formulations that release appropriate amounts of drug at the target site may be useful for superficial fungal infections. However, antifungal drugs are known to show high affinity to keratin, which reduces nail penetration and efficacy because keratin binding can decrease drug penetration to deeper layers of the nail, even after repeated application. Therefore, safe and efficacious topical formulations with greater nail plate penetration and clinical efficacy, and fewer side effects, are required.

Efinaconazole ((2 R,3R)-2-(2,4-difluorophenyl)-3-(4-methylenepiperidin-1-yl)-1-(1H-1,2,4-triazol-1-yl) butan-2-ol; EFN) is a novel triazole antifungal that inhibits the fungal cytochrome P450 enzyme (lanosterol-14α demethylase, CYP51) and blocks fungal membrane ergosterol biosynthesis, thereby disrupting the membrane integrity and growth of fungi (Jo Siu et al., 2013; Glynn et al., 2015). Moreover, azoles have higher affinity to fungal CYP51 than the mammalian enzymes. EFN is the first azole drug approved by the US Food and Drug Administration (FDA) for topical treatment of onychomycosis in the USA (Lipner & Scher, 2015); EFN has the unique property of relatively lower binding to keratin, such that bound EFN is released more rapidly from keratin; this promotes the nail penetration of EFN. However, to increase complete cure rates, a topical formulation with enhanced efficacy and penetration speed after application is still required.

To address the problems of drug solubility and stability, as well as improve the efficacy of conventional antifungal agents, different strategies have been utilized, such as chemical modification of antifungal agents, carrier-based delivery using penetration enhancers, preparation and application of nanoformulations, and combined treatment with physical methods to enhance drug delivery, including iontophoresis, photodynamic therapy, and lasers (Dutet & Delgado-Charro, 2009; Eichner et al., 2017). However, the composition of topical solutions may significantly affect drug release and skin and nail permeability (Brown et al., 2009). In addition, formulations containing active ingredients of triazole exhibit varying degrees of instability and solubility in solution during storage. Certain formulations are known to exhibit discoloration within storage periods as short as 1 or 2 days, resulting in solutions ranging in color from yellow to deep red or brown. This can discourage the use of prescribed formulations by patients.

To date, the effectiveness of delivery of topical antifungals for onychomycosis has been limited due to their short residence times and hydrophobic nature. Therefore, recent studies focused on the development of topical solutions containing permeation enhancers, nail lacquer, and films or adhesive patches, which may enhance nail hydration and residence time (Tiwary & Sapra, 2017). Solid lipid nanoparticles, nanostructured lipid carriers, liposomes, and ethosomes for systemic and local nail and skin infections have also been studied (Moazeni et al., 2016; Trombino et al., 2016). Furthermore, different physical and chemical methods have been proposed to enhance topical drug delivery to nails (Brown et al., 2009; Dutet & Delgado-Charro, 2009; Nogueiras-Nieto et al., 2011). Recently, Rocha et al. prepared voriconazole-loaded nanostructured lipid carriers and evaluated drug penetration of porcine hooves *in vitro*; the utility of urea as a permeation enhancer was also evaluated. The study demonstrated higher encapsulation efficiency, along with significantly enhanced drug penetration into the deeper regions of the hooves, in comparison to the unloaded drug, which was attributed to the presence of urea as a permeation enhancer (Rocha et al., 2017). Another study, by Naumann et al., compared four different formulations (colloidal carrier system, nail lacquer, solution, and hydrogel) of the novel antifungal agent, EV-086K, for treatment of onychomycosis. Enhanced drug delivery and penetration potential were demonstrated for the colloidal system compared to the other formulations (Naumann et al., 2014).

The main objective of the present study was to design a topical delivery system for EFN with highly penetrability, rapid absorption, and high stability during storage. To enhance the skin and nail permeability of EFN, Transcutol P and isopropyl myristate were used as penetration enhancers, along with cyclomethicone as a wetting agent. In addition, ethanol was used as a volatile solvent to enhance the solubility of EFN. Fast infiltration and permeation of EFN through human skin and nail plate were expected due to the enhanced solubility, effects of permeation enhancers, and lower binding of the drug to keratin. In addition, the stability of EFN topical solution during storage was improved by incorporation of an antioxidant, chelating agent, and pH-adjusting agent. After assessing the artificial membrane permeability of EFN using different topical solutions, the stability at 65 °C and *in vitro* antifungal activity were evaluated. The skin permeability, nail permeation, and infiltration of EFN were then evaluated using the selected topical EFN formulations (EFN-K). Finally, the *in vivo* antifungal efficacy of the selected EFN topical formulation was assessed in a *T. mentagrophytes*-infected guinea pig onychomycosis model.

## 2. Materials and methods

### 2.1. Materials

EFN was obtained from Cipla Limited (Mumbai, India). Transcutol P was obtained from Gattefosse (Saint-Priest, France). Quercetin (purity > 95%, internal standard, IS), isopropyl myristate, butylated hydroxytoluene (BHT), butylated hydroxyanisole (BHA), ethylenediaminetetraacetic acid (EDTA), bovine serum albumin (BSA), polyoxyethylene (20) oleyl ether (Brij O20), sodium azide, sorbic acid, citric acid, cycloheximide, and chloramphenicol were obtained from Sigma-Aldrich Inc. (St. Louis, MO, USA). Diethylenetriaminepentaacetic acid (DTPA) was purchased from Thermo Fisher Scientific (Waltham, MA, USA). Jublia^®^ (commercial 10% EFN topical solution, reference control #1) was obtained from Valeant Pharmaceuticals International Inc. (Laval, QC, Canada). Fulcare^®^ (8% ciclopirox nail lacquer, reference control #2) was purchased from A. Menarini Industrie Farmaceutiche Riunite S.r.l. (Firenze, Italy). Sabouraud’s dextrose agar (SDA) medium and yeast malt (YM) agar medium were obtained from BD Biosciences (San Jose, CA, USA). Solvents for high-performance liquid chromatography (HPLC) were obtained from Merck Millipore (Billerica, MA, USA) and Thermo Fisher Scientific Inc.

### 2.2. Animals

Hartley strain guinea pigs (males, 300–400 g) were purchased from Saeronbio (Uiwang, Republic of Korea). Ethical approval for this study was obtained from the Institutional Animal Care and Use Committee (IACUC) of Chung-Ang University (Seoul, Republic of Korea). All animal experiments were performed in accordance with the National Institutes of Health Guidelines for the Care and Use of Laboratory Animals and the IACUC guidelines.

### 2.3. Preparation and *in vitro* artificial transdermal membrane permeability

The EFN-loaded topical formulations (10%, w/w) developed in this study are listed in Table 1 and were prepared using a microfluidization method. Briefly, EFN (5 g) was dissolved in 20 g of ethanol, and cyclomethicone, Transcutol P, and isopropyl myristate were added to the EFN solution with three different amounts of cyclomethicone (10%, 13%, or 15%), Transcutol P (10%, 15%, or 20%), and isopropyl myristate (6%, 15%, or 20%). Then, 200 mg of aqueous solution of EDTA (0.025 mg/mL), 50 mg of sorbic acid, and 50 mg of BHA were added to the mixture as a chelating agent, antioxidant, and pH-adjuster, respectively. After mixing for 10 minutes, the EFN topical solution was passed through a microfluidizer (LM20; Microfluidics, Westwood, MA, USA) at a pressure of 15,000 psi for 15 cycles to form a clear solution. Finally, the weight of topical solution was maintained at 100% by addition of ethanol.

**Table 1.** Compositions of the topical formulations of EFN.

Ingredients (%, w/w)	EFN-A	EFN-B	EFN-C	EFN-D	EFN-E	EFN-F	EFN-G	EFN-H	EFN-I	EFN-J	EFN-K
EFN	10	10	10	10	10	10	10	10	10	10	10
Ethanol	53.8	56.8	51.8	55.8	50.8	45.8	59.8	50.8	45.8	51.8	59.8
Cyclomethicone	13	10	15	13	13	13	13	13	13	15	13
Transcutol P	12	12	12	10	15	20	10	10	10	12	10
Isopropyl myristate	10	10	10	10	10	10	6	15	20	10	6
EDTA disodium/DTPA	0.00025	0.00025	0.00025	0.00025	0.00025	0.00025	0.00025	0.00025	0.00025	0.00025	0.00025
BHT/BHA	0.1	0.1	0.1	0.1	0.1	0.1	0.1	0.1	0.1	0.1	0.1
Purified water	1	1	1	1	1	1	1	1	1	1	1
Sorbic acid/Citric acid	0.1	0.1	0.1	0.1	0.1	0.1	0.1	0.1	0.1	0.1	0.1
Total (%)	100	100	100	100	100	100	100	100	100	100	100

EFN: efinaconazole; EDTA: ethylenediaminetetraacetic acid; DTPA: diethylenetriaminepentaacetic acid; BHT: butylated hydroxytoluene; BHA: butylated hydroxyanisole.

Next, to compare the skin permeability of the EFN topical solutions, *in vitro* transdermal membrane permeability through a synthetic membrane (Strat-M^®^; EMD Millipore, Temecula, CA, USA) was evaluated using a Franz diffusion cell system (Labfine, Gyeonggi-do, Republic of Korea). Each Strat-M^®^ disc (25 mm in diameter) was mounted on the receiver compartment with a diffusion area of 0.785 cm^2^. After assembling the donor compartment, 5 mL of phosphate-buffered saline (PBS; pH 7.4) containing 1.5% Brij O20 and 0.1% sodium azide was added to the receiver compartment, and the cells were allowed to equilibrate for at least 60 minutes to confirm membrane hydration. After loading, 127 µL/cm^2^ of each 10% EFN topical solution was applied to the donor compartment. The receiver compartment was stirred at 600 rpm in a heating system to maintain a membrane surface temperature of 32 °C throughout the experiment. Then, 300 μL of the receptor phase was withdrawn after 12 and 24 hours, and each aliquot was replaced with the same volume of fresh PBS (pH 7.4) containing 1.5% Brij O20 and 0.1% sodium azide. The collected samples were then filtered through a membrane filter (0.45 µm, polyvinylidene fluoride, PVDF) and the concentration of EFN that permeated through the artificial membrane was determined by HPLC with a C18 column (150 × 4.6 mm, 5 µm, 100 Å; 20-µL sample injection) at 30 °C. EFN was measured using an ultraviolet (UV) detector at 205 nm by the isocratic elution method with acetonitrile:water (64:36, v/v) as the mobile phase at a flow rate of 1 mL/min.

### 2.4. Stability studies

The results of the artificial membrane permeability tests informed the compositions of the permeation-enhancing agents used in the EFN topical solution; furthermore, the antioxidant and chelating agents included in the selected formulations (EFN-C and EFN-G) were modified to improve drug stability. After selecting the EFN topical formulations, EFN-J and EFN-K were stored at 65 °C ± 2 °C for an accelerated stability test. Physicochemical properties such as phase separation and precipitation, as well as physical appearance and drug contents, were analyzed weekly. In addition, discoloration of the EFN topical solutions was observed by measuring the absorbance values at 400, 500, and 600 nm; the acceptable absorbance values for drug stability were considered to be less than 0.4, 0.1, and 0.1 AU at 400, 500, and 600 nm, respectively.

### 2.5. *In vitro* antifungal activity

The antifungal activities of EFN-J and EFN-K were evaluated by disk diffusion assay and compared to reference control #1 (commercial 10% EFN topical solution). Briefly, *T. rubrum* (ATCC^®^ MYA-4438), obtained from the American Type Culture Collection (ATCC, University Boulevard, Manassas, VA, USA), was cultured in SDA medium containing chloramphenicol (20 µg/mL) and cycloheximide (200 µg/mL) at 25 °C for 5 days. After incubation, the concentration of spores in the suspension was adjusted with 0.1 M PBS to 1 × 10^8^ CFU/mL using a hemocytometer. Next, a 1-cm-dimaeter hole was punched in sterilized filter paper, which was then laid on the center of each SDA plate followed by uniform spreading of 100 µL of the prepared spore suspension on the plate. Each disk was then impregnated with 5 µL of sample solution. Control plates were prepared by incubating a disk soaked with sterilized water and spore suspension. After incubating at 25 °C for 14 days, the zone of inhibition (ZOI) was calculated by measuring the mean diameter of the area of growth inhibition around the disk.

### 2.6. *In vitro* permeability across human skin

To compare the skin permeability of the selected EFN topical solution with the reference control solution, an *in vitro* human skin permeability study was performed using the Franz diffusion cell system (Labfine). Excised full-thickness human skin (HansBiomed Corp., Daejeon, Republic of Korea) was mounted with the stratum corneum (SC) facing upward on the acceptor compartment of a Franz diffusion cell system, to which 5 mL of PBS (pH 7.4) containing 1.5% Brij O20 and 0.1% sodium azide was then added. Next, the donor compartment was clamped in place (diffusion area, 0.785 cm^2^) and the cells were allowed to equilibrate for at least 60 minutes to confirm the integrity of the skin. After reaching equilibrium, 127 µL/cm^2^ of each 10% EFN topical solution was applied to the donor compartment. The acceptor compartment was stirred at 600 rpm and heated to maintain a skin surface temperature of 32 °C throughout the experiment. Then, aliquots of 300 µL of sample solution were collected at predetermined time intervals (1, 3, 6, 9, 12, and 24 hours) from each diffusion cell and replaced with the same volume of fresh PBS (pH 7.4) containing 1.5% Brij O20 and 0.1% sodium azide. The collected samples were then filtered through a membrane filter (0.45 µm, PVDF), and the amount of EFN that permeated through the full-thickness human skin was determined by HPLC at 205 nm by the isocratic elution method, as described above.

### 2.7. *In vitro* human nail deposition and penetration

To evaluate the infiltration of EFN topical solution across human nail, the *in vitro* nail permeation and deposition were assessed using a Franz diffusion cell system. Human fingernails from different adult human cadavers were obtained from Science Care, Inc. (Phoenix, AZ, USA) and kept in a closed container at −70 °C until use. One day before the experiment, nail plates were removed from a deep freeze (−70 °C) and kept at room temperature overnight. On the day of the experiment, nail plates were hydrated in normal saline for 3 hours at room temperature. The hydrated nails were firmly fixed between two Teflon gaskets of the nail holder in the donor compartment, which was then mounted and clamped onto the receptor compartment filled with PBS (pH 7.4) containing 4% (w/v) BSA and 0.01% (w/v) sodium azide (diffusion area, 28.27 mm^2^). Next, the donor compartment was clamped in place; the cells were allowed to equilibrate for at least 60 minutes and were stirred at 600 rpm in an incubator at 32 °C. For the permeation study, 1.77 µL/mm^2^ of 10% EFN solution was applied to the human nail plate in the donor compartment once daily for 7 days, and the nail plate was washed with distilled water between doses. On day 1 after loading, 300 µL aliquots of the samples from each receptor compartment were withdrawn at different time points (1, 2, 4, 6, 8, 20, and 24 hours) and replaced with the same volume of fresh PBS (pH 7.4) containing 4% (w/v) BSA and 0.01% (w/v) sodium azide. From day 2, the receptor phase solution was withdrawn every 24 hours and replaced with the same volume of fresh PBS (pH 7.4) containing 4% (w/v) BSA and 0.01% (w/v) sodium azide. The withdrawn samples were stored at −70 °C.

Furthermore, the nail-deposited EFN formulations were monitored for 24 hours after dosing. After 24-hour exposure to 50 µL of 10% EFN topical solution, the diffusion cells were disassembled and the nails were removed from the chamber, followed by washing twice with 5 mL of deionized water. Then, the nail area exposed to the formulation was cut and dissolved in 1 N sodium hydroxide:methanol (1:1, v/v) solution. The nail samples were then incubated at 60 °C for 4 hours, followed by the addition of 1 mL of methanol and 1 mL of ethanol with continuous vortex mixing. The mixture was centrifuged at 4,000 rpm for 10 minutes, and the clear supernatant was then withdrawn and evaporated using a centrifugal evaporator (Genevac Ltd., Ipswich, UK). Next, the dried residue was resuspended with 100 µL of a 1:1 (v/v) mixture of ethanol and mobile phase (acetonitrile:water, 64:36, v/v). The concentrations of EFN in the nail, as well as the receptor phase solution, were determined using an Agilent 6120 quadruple liquid chromatography/mass spectrometry (LC/MS) system with a Phenomenex Luna C18 column (150 × 4.6 mm, 5 µm), and the samples were chromatographed using isocratic mobile phase at a flow rate of 1 mL/min. Aliquots of 20 µL were injected and EFN and quercetin were measured in positive and negative ionization modes, respectively, using an atmospheric pressure ionization-electron spray (API-ES) source. The following parameters were optimized for EFN analysis: capillary voltage, 3.0 kV; drying gas flow rate, 12.0 L/min; and drying gas temperature, 304 °C. The fragment ions at this capillary voltage were detected and quantified at ([M + H]^+^ = 349.3) and ([M + H]^+^ = 301.0) for EFN and quercetin, respectively.

### 2.8. *In vivo* antifungal efficacy

To evaluate the antifungal efficacy of EFN-J and EFN-K, onychomycosis was induced in guinea pigs by infecting their paw nails with *T. mentagrophyte*s (ATCC 18748). After incubation of *T. mentagrophytes* at 25 °C for 7 days in YM agar media, a spore suspension was prepared at a concentration of 1 × 10^8^ CFU/mL in YM broth. Then, the hind paw nails of guinea pigs were covered with sterilized gauze wetted with 100 μL of the *T. mentagrophytes* spore suspension, three times a week for 4 weeks. To increase the infection efficiency, the animals’ legs were sealed with latex socks for 4 weeks. The guinea pigs were randomly divided into five treatment groups (six guinea pigs per group). A daily 1 μL/mm^2^ topical treatment of vehicle control (topical solution of EFN-K without drug), reference control #1 (commercial 10% EFN topical solution), reference control #2 (commercial 8% ciclopirox nail lacquer), EFN-J (10% EFN) or EFN-K (10% EFN) was applied for 4 weeks using a nail brush on all hind paw nails of each animal (six hind paw nails per guinea pig). To promote evaporation of the test solutions, the hind paws were held stable for 1 minute after application. Non-infected guinea pigs were treated as negative controls to allow comparison with normal paw nails. After infection followed by 4 weeks of treatment, to directly monitor and assess the antifungal effect, the nails from the guinea pigs were collected, homogenized in PBS (pH 7.4) containing 0.25% trypsin and 10 mmoL/L FeCl_3_, and digested at 37 °C for about 1 hour. After diluting with 0.1 M PBS, the nail samples were inoculated into YM agar medium containing 200 μg/mL cycloheximide and 20 μg/mL chloramphenicol, and cultured at 25 °C. After 14 days of incubation, viable fungal colonies were used as an index of the nail penetration and therapeutic efficacy of EFN-K for treatment of onychomycosis. Furthermore, the collected nails were stained with periodic acid-Schiff (PAS) for histological examination of fungal infection. Briefly, the nails were biopsied and fixed with 10% neutral buffered formalin for 1 day, and embedded in paraffin. Paraffin-embedded nail blocks were then sliced to a thickness of 3 μm, stained with PAS, and imaged under light microscopy. The remaining nails from each guinea pig were homogenized, diluted with 0.1 M PBS and inoculated into YM agar medium with 200 μg/mL of cycloheximide and 20 μg/mL of chloramphenicol. After 14 days of incubation at 25 °C, the living fungal colonies were counted to confirm the nail-penetrating and therapeutic effects of EFN-J and EFN-K for the treatment of onychomycosis.

### 2.9. Statistical analysis

Unpaired data were evaluated using Student’s *t-*test for comparisons between two mean values, and one-way analysis of variance (ANOVA) followed by Tukey’s multiple-comparison test for comparisons between more than two mean values. All data are expressed as means ± standard deviation. In all analyses, *p* < .05 was taken to indicate statistical significance.

## 3. Results and discussion

### 3.1. Preparation and characterization of EFN topical solution

Based on the solubility of EFN and the known skin permeation-enhancing activity of ethanol, ethanol was selected as the drug delivery vehicle (Lachenmeier, 2008; Ng, 2018). Cyclomethicone was selected as the wetting agent due to its varying rates of evaporation, low viscosity, and non-greasy feel. In addition, it showed miscibility with the other excipients used in the topical formulations. Drug delivery across the nail plate, as well through the skin barrier, is a major challenge. Therefore, to enhance transungual permeability, isopropyl myristate and Transcutol P were added to the topical solution as lipophilic permeation enhancers. Isopropyl myristate has a disordering effect on the rigid subcutaneous lipid membranes (Eichner et al., 2017). Furthermore, a recent study demonstrated the permeation-enhancing activity of Transcutol P through the bovine hoof membrane, which is structurally similar to human nails, via conformational changes in the keratin structure (Bseiso et al., 2016). To improve the stability of the prepared formulations, EDTA or DTPA, BHA or BHT, and sorbic acid or citric acid were used as a chelating agent, antioxidant, and pH-adjusting agent, respectively. Edetate salts, such as sodium EDTA and DTPA, as well as citric acid and sorbic acid possess antimicrobial activity and show synergistic effects when used in combination (Heydari et al., 2013). Based on the formation of a clear solution, we further assessed the *in vitro* permeability of each formulation across an artificial transdermal membrane, to optimize each component.

### 3.2. *In vitro* artificial transdermal membrane permeability of EFN topical solutions

**Figure 1.**
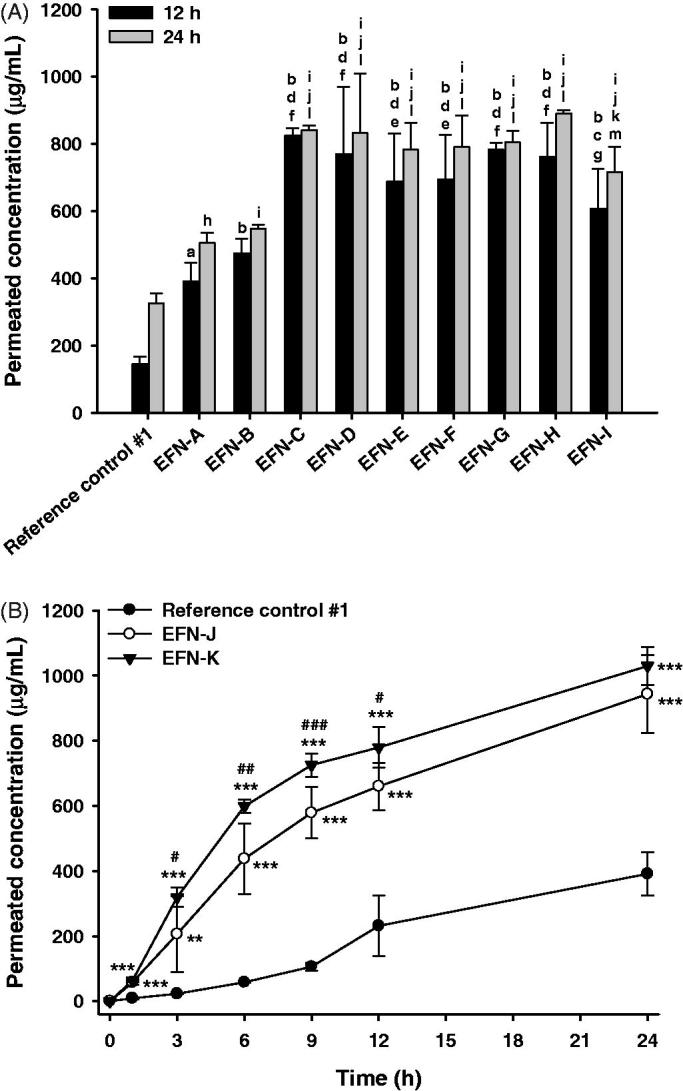
*In vitro* artificial transdermal membrane permeability of the efinaconazole (EFN)-dissolved topical formulations. (A) The cumulative permeated concentrations of EFN at 12 and 24 h after topical EFN application. Each value represents the mean ± standard deviation (*n* = 6). ^a^*p* <.01, ^b^*p* <.001 compared to the reference control #1 at 12 h. ^c^*p* <.05, ^d^*p* <.001 compared to the EFN-A at 12 h. ^e^*p* <.05, ^f^*p* <.001 compared to the EFN-B at 12 h. ^g^*p* <.05 compared to the EFN-C at 12 h. ^h^*p* <.01, ^i^*p* <.001 compared to the reference control #1 at 24 h. ^j^*p* <.001 compared to the EFN-A at 24 h. ^k^*p* <.05, ^l^*p* <.001 compared to the EFN-B at 24 h. ^m^*p* <.01 compared to the EFN-H at 24 h. (B) Time course of cumulative EFN permeation concentrations for reference control #1, EFN-J, and EFN-K. Each value represents the mean ± standard deviation (*n* = 6). **p <.01, ***p < .001 compared to the reference control #1. ^#^*p* <.05, ^##^*p* <.01, ^###^*p* <.001 compared to the EFN-J.

The *in vitro* permeabilities of nine EFN topical solutions (EFN-A − EFN-I) and the reference control #1 through the artificial transdermal membrane were evaluated at 12 and 24 hours after loading the samples (Figure 1(A)). The membrane permeability of EFN topical solutions was significantly greater compared to reference control #1 (commercial 10% EFN topical solution), due to the addition of Transcutol P and isopropyl myristate. In addition, when the percentage of wetting agent (cyclomethicone) in the topical solution was decreased by 2% (EFN-B) compared to EFN-A, no significant improvement in permeability was observed at 12 or 24 hours, whereas increasing the proportion of cyclomethicone in the formulation by 2% increased the artificial membrane permeability of EFN-C by 1.68- and 2.57-fold compared to those of the EFN-A and reference control, respectively, after 24-hour incubation. Cyclomethicone is a silicone-based oil that acts as a mild water repellent with antistatic, emollient, humectant, and viscosity-controlling functions (Pellicoro et al., 2013). The presence of cyclomethicone was reported to result in low surface tension in cosmetic formulations and thus good spreadability (Bhatt & Pillai, 2015). The low surface tension induced by cyclomethicone is expected to improve EFN infiltration into difficult-to-reach crevices in the nail bed, as well as the air pockets that are often present in onychomycosis.

Next, to evaluate the effects of Transcutol P on the infiltration of EFN through the artificial skin membrane, we decreased the proportion of Transcutol P by 2% (EFN-D), or increased it by 3% (EFN-E) or 8% (EFN-F), with respect to EFN-A. Although the cumulative infiltrated concentration of each formulation (EFN-D − EFN-F) was higher than that of EFN-A, no significant differences in permeability were observed among them. In addition, due to the various advantages of Transcutol P, such as its solubilizing activity, permeation enhancement, colorless and nearly odorless nature, non-volatility, non-toxicity, biocompatibility with skin, and miscibility with polar and non-polar solvents, it has been used in topically applied FDA-approved products at proportions up to 49.9% (Mura et al., 2000; Osborne & Musakhanian, 2018). We investigated the effects of isopropyl myristate on the membrane infiltration of EFN topical solutions (EFN-G − EFN-I). All three formulations showed significantly enhanced membrane permeability compared to the reference control #1 and EFN-A, whereas the effect of isopropyl myristate on the artificial permeability of EFN was not significant, suggesting that the increase in permeability of EFN may not be directly related to the amount of isopropyl myristate; instead, it may be due to the combined effects of isopropyl myristate and ethanol (Karande & Mitragotri, 2009). However, additional permeability studies using the artificial transdermal membrane are required to determine the time course of drug saturation. Measurement of penetrated EFN concentration at early stages (<12 h) will confirm the effects of Transcutol P or isopropyl myristate on the penetration of EFN through the artificial transdermal membrane.

Based on the results of studies on artificial transdermal membrane permeability, the optimum EFN topical solutions were EFN-C and EFN-G; accordingly, these were selected for further study. However, EFN-C and EFN-G showed discoloration after storage for 1 month at 65 °C. Therefore, we replaced the antioxidant, chelating agent, and pH-adjusting agent in EFN-C with BHT, DTPA, and citric acid, respectively. As a result, the infiltration of EFN from EFN-J or EFN-K through an artificial transdermal membrane was significantly improved compared with reference control #1 (Figure 1(B)). EFN was significantly penetrated at 1 h after loading of EFN-J or EFN-K, whereas EFN was rarely permeated until 1 hour after loading of reference control #1. After 6 h, the cumulative infiltrated EFN concentration from EFN-J and EFN-K was 409 ± 109 and 598 ± 20.1 µg/mL, respectively, which was 7.50- and 10.3-fold greater than reference control #1. The permeated concentrations of EFN from EFN-J and EFN-K after 12 h were still 2.85- and 3.36-fold higher than that of EFN from the reference control #1, respectively. Furthermore, compared to reference control #1, the flux values for EFN-J and EFN-K were increased by 250- and 273-fold, respectively. These results support a synergistic role for Transcutol P and isopropyl myristate in the formulation. Moreover, the drug contents of the selected EFN topical solutions (EFN-J and EFN-K) were maintained at >98%. There was no precipitation, phase separation, or discoloration after 1 month of storage at 65 °C ± 2 °C (data not shown). In addition, the UV absorbance values of all three formulations remained within acceptable limits for up to 5 weeks (<.07 AU) (Figure S1).

### 3.3. *In vitro* antifungal efficacy of EFN topical solution

**Figure 2.**
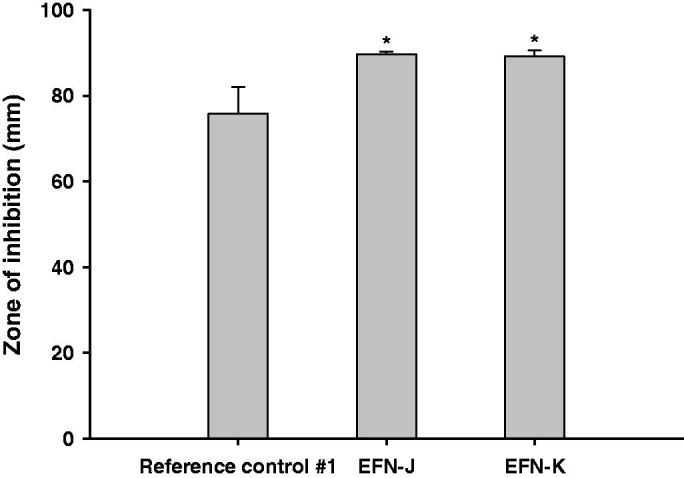
*In vitro* antifungal efficacy of the EFN topical solutions. Zones of inhibition for the reference control #1 (commercial 10% EFN topical solution), EFN-J, and EFN-K solutions by disk diffusion assay. Each value represents the mean ± standard deviation (*n* = 4). *p <.05 compared to reference control #1.

The *in vitro* antifungal activities against *T. rubrum* of EFN-J and EFN-K were compared to those of the reference control #1 using the disk diffusion assay test method (Figure 2). After 14 days of incubation of *T. rubrum* with the drug, the ZOI values of reference control #1, EFN-J, and EFN-K were 75.8 ± 6.29, 89.7 ± 0.577, and 89.2 ± 1.14 mm, respectively. Furthermore, the antibacterial efficiencies of EFN-J and EFN-K were both 1.18-fold higher than that of reference control #1. These improvements in antifungal activity of EFN-J and EFN-K against *T. rubrum* may have been due to the presence of permeation enhancers, which cause rapid penetration, increased uptake by endocytosis, as well as passive diffusion of EFN into the cytoplasm (Tian et al., 2017). In addition, these results also indicated that EFN-J and EFN-K have better stability and efficiency compared to reference control #1 during incubation at room temperature, where the most important factor in the antifungal effect of a formulation is its EFN content. All of the solutions contained the same proportion of EFN (10%) at the manufacturing step; therefore, antioxidant and chelating agents, such as DTPA and BHT, in the EFN-J and EFN-K may have improved their stability.

### 3.4. *In vitro* human skin permeability of EFN topical solutions

**Figure 3.**
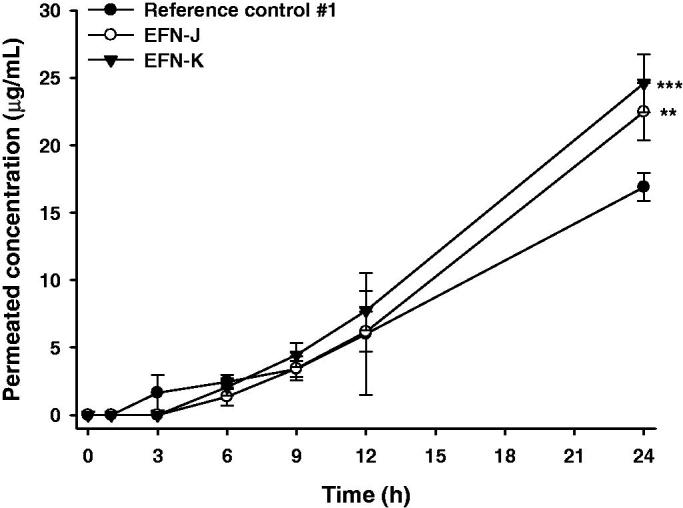
*In vitro* permeation through full-thickness human skin. Time course of cumulative EFN permeation concentrations for the reference control #1, EFN-J, and EFN-K solutions. Each value represents the mean ± standard deviation (*n* = 4). **p <.01, ***p <.001 compared to reference control #1.

The improvements in skin infiltration of EFN-J and EFN-K through human skin compared to the reference control #1 are shown in Figure 3. EFN permeation into the receiver compartment was observed at 3 hours after loading reference control #1, whereas EFN-J and EFN-K rarely permeated through the skin for up to 3 hours. However, after 24 hours of incubation, the levels of infiltrated EFN from EFN-J and EFN-K were 1.33- and 1.46-fold higher than that of reference control #1. In addition, the skin flux of EFN-K was 2.06-fold greater than that of reference control #1. The enhanced skin infiltration of EFN-K may have been mediated by the generation of a supersaturated secondary formulation of EFN after evaporation of ethanol; thereafter, the EFN in combination with Transcutol P and isopropyl myristate effectively increased the penetration of EFN across the SC, leading to a higher degree of skin permeation. The presence of Transcutol P in a formulation can increase the thermodynamic driving force, facilitate partitioning of the drug, and maintain hydrated dynamics in the SC and intercellular lipid fluidization (Osborne & Musakhanian, 2018). Recent studies also suggested a permeation-enhancing action of isopropyl myristate, which mimics skin lipids due to its polar and nonpolar properties (Ottaviani et al., 2006; Ilbasmis-Tamer et al., 2017). This result indicated that EFN-K has superior antifungal efficacy due to enhanced and sustained delivery of EFN into the distal and lateral nail fold, nail bed, and eponychium. However, further permeability studies using lower volumes of EFN topical solutions (<10 µL/cm^2^) are required to determine the clinical relevance of the skin permeability of EFN topical solution at a finite dose.

### 3.5. *In vitro* nail permeation of EFN topical solutions

**Figure 4.**
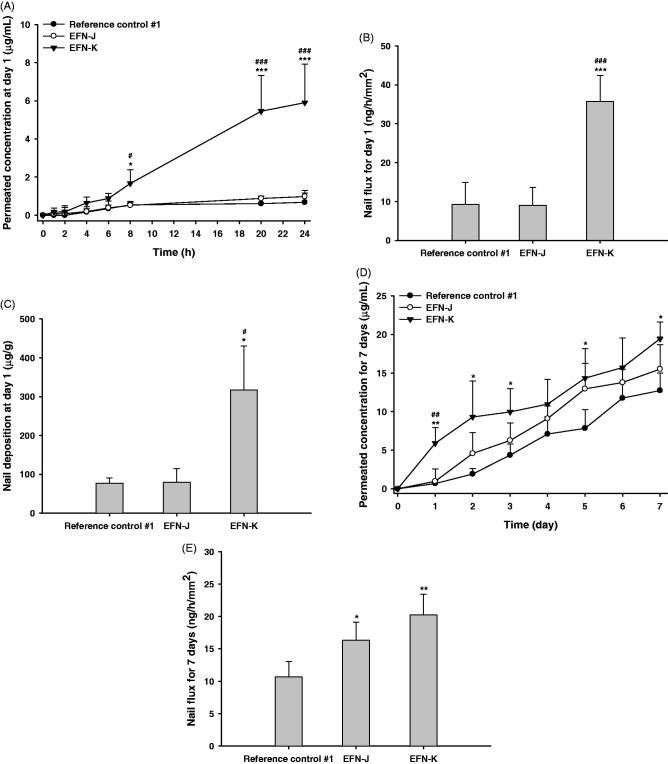
*In vitro* permeation through human nail plates. Time course of cumulative EFN permeation (across the nail plate) concentrations (A), nail flux of EFN (B), and infiltrated EFN concentrations in the nail plate (C) at 1 day after application of the reference control #1, EFN-J, and EFN-K solutions. Time course of cumulative EFN permeation (across the nail plate) concentrations (D) and nail flux of EFN (E) at 7 days after once-daily application of the reference control #1, EFN-J, and EFN-K solutions. Each value represents the mean ± standard deviation (*n* = 4). *p <.05, **p <.01, ***p <.001 compared to reference control #1. ^#^*p* <.05, ^##^*p* <.01, ^###^*p* <.001 compared to the EFN-J.

The permeation potential across the nail plate of EFN in the reference control #1, EFN-J, and EFN-K was evaluated as shown in Figure 4. At 1 hour after drug loading, the permeability of EFN from EFN-K was 0.170 ± 0.202 µg/mL, which was 2.75-fold higher than that of EFN-J, whereas reference control #1 did not permeate across the nail plate for up to 2 hours. These observations confirmed the rapid penetration of EFN-K compared to reference control #1 and EFN-J. In addition, the level of permeated EFN in the receiver compartment was markedly increased up to 8 hours after loading with EFN-K compared to the other formulations. After 8 hours, the cumulative EFN concentration in EFN-K that had penetrated through the human nail plate was 3.21- and 3.05-fold higher than those of EFN-J and reference control #1, respectively. Furthermore, after 24-hour incubation, the level of EFN in the receiver compartment reached 5.91 ± 2.02 µg/mL for EFN-K, which was 6.02- and 8.69-fold greater than those of EFN-J and reference control #1, respectively (Figure 4(A)). This increased the nail flux of EFN-K at day 1 to 35.7 ± 6.74 ng/h/mm^2^, which was 3.87-fold higher than that of reference control #1 (9.23 ± 5.68 ng/h/mm^2^) (Figure 4(B)). EFN-J exhibited similar nail flux on day 1 to reference control #1. In addition, its skin permeability was equivalent to that of ENF-K, but it did not markedly increased nail permeability compared to EFN-K; this may have been due to the differences in lipid levels between the skin and nail; one of the major differences in morphology between human skin and nail is the low level of lipids in the nail matrix (<5% in human nail vs. approximately 15% in the SC), such that the nail plate is more hydrophilic than the skin layer (Bhatt & Pillai, 2015; Matsuda et al., 2016). EFN-J, which contains more hydrophobic permeation enhancers than EFN-K, showed opposite permeation profiles across human skin and nail plate because lipids are more susceptible to the disruptive effects of permeation enhancers, such as Transcutol P and isopropyl myristate. This was also shown by the deposition of EFN in the nail plate on day 1 after treatment with EFN-J. As illustrated in Figure 4(C), EFN-K penetrated the human nail plate more effectively than the reference control or EFN-J, such that the level of infiltrated EFN was 4.11- and 3.99-fold greater than for reference control #1 and EFN-J, respectively.

After 7 days of application of EFN-J, EFN had significantly permeated through the nail plate, in an amount 1.22-fold greater than that of reference control #1; this resulted in a 1.53-fold increase in the nail flux of EFN-J, suggesting that it takes 24 hours to form a concentration gradient across channels within the nail plate filled with the second formulation (Figure 4(D)). On day 7, the cumulative penetrated concentration of EFN from EFN-K was 19.5 ± 2.19 µg/mL. Moreover, the nail flux values at 7 days of treatment with EFN-K were increased by 89.7% and 23.8% compared to reference control #1 and EFN-J, respectively (Figure 4(E)).

Based on the overall results, it appears that markedly enhanced skin and nail penetration of EFN-K are induced by low surface tension after topical application, as well as maintenance of sufficient solubility of EFN in the secondary formulation after evaporation of ethanol; this allowed EFN to penetrate through the nail plate and into the nail bed, by spreading under the nail plate and filling the air gap at the site of infection, and supplying a sufficient concentration of drug to the diseased tissues after topical application.

### 3.6. *In vivo* antifungal activities of EFN topical solutions in the *T. mentagrophytes*-infected guinea pig model

**Figure 5.**
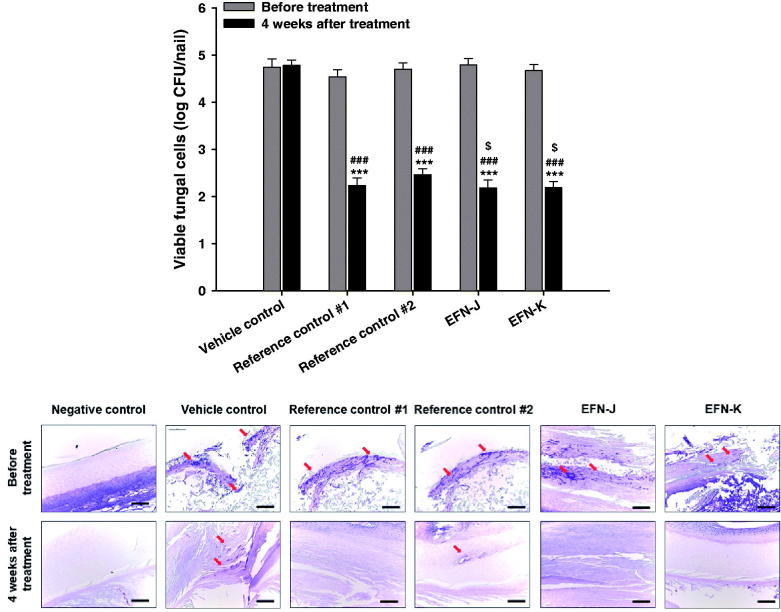
*In vivo* antifungal effects of EFN topical solutions in the *T. mentagrophytes*-infected onychomycosis guinea pig model following once-daily treatment with vehicle control (topical solution of EFN-K without drug), reference control #1 (commercial 10% EFN topical solution), reference control #2 (commercial 8% ciclopirox nail lacquer), EFN-J (10% EFN), and EFN-K (10% EFN). (A) Viable fungal cell counts in guinea pig nails (log CFU/nail) before and after 4 weeks of treatment. Each value represents the mean ± standard deviation (*n* = 6). ***p <.001 compared to the viable fungal cell count before treatment with each material. ^###^*p* <.001 compared to the viable fungal cell count in the vehicle control group after 4 weeks of treatment. ^$^*p* <.05 compared to the viable fungal cell count in the reference control #2 after 4 weeks of treatment. (B) Representative light micrographs of sections from guinea pig nails stained with periodic acid-Schiff (PAS) before and 4 weeks after treatment. Scale bar represents 100 µm.

Next, we investigated the curative effects of the antifungal activity of reference controls, EFN-J, and EFN-K on onychomycosis in guinea pigs. As shown in Figure 5(A), all guinea pig paw nails were significantly infected by *T. mentagrophytes*, and before drug treatment, the mean viable fungal cell counts in the vehicle control, reference control #1, reference control #2, EFN-J, and EFN-K groups were 4.74 ± 0.173, 4.53 ± 0.155, 4.70 ± 0.137, 4.79 ± 0.132, and 4.67 ± 0.129 log CFU/nail, respectively. After 4 weeks of treatment, the number of colonies in the infected nails treated with vehicle solution was not significantly reduced. In contrast, the number of viable fungal colonies of reference control #1 was decreased by approximately 50.8% compared to the initial value, which resulted in a 6.33% decrease in viable fungal colonies compared to the reference control #2 group. Furthermore, EFN-J and EFN-K also markedly inhibited the growth of *T. mentagrophytes* present in the keratin layer, resulting in decreases of 54.5% and 53.2% in the mean viable fungal cell counts compared to their initial values, respectively. At 4 weeks after treatment, the antifungal activities of EFN-J and EFN-K were still superior to the vehicle-treated and reference control #2 groups, and the reductions in number of living fungal colonies were 12.7% and 10.5% greater than reference control #2, respectively.

Regarding the histological evaluation of infected paw nails from guinea pigs treated with vehicle, reference controls, EFN-J, and EFN-K, PAS staining results showed similar trends (Figure 5(B)). The negative control group (non-infection) showed no positively stained areas, while the other groups were shown to be infectious by areas of positive staining (red arrow) at 4 weeks after infection. Consistent with this, at 4 weeks after treatment with each topical solution, the injected fungi continued to appear in the vehicle control group (no drug treatment), while in the reference control #1, EFN-J, and EFN-K groups nail regions were clear; this indicated that the structure of keratin, which had been denatured, was restored to normal (Figure 5(B)).

In this study, all of the 10% EFN topical solutions showed more effective antifungal activity in *T. mentagrophytes*-infected nails than reference control #2 (8% ciclopirox nail lacquer), which may have been due to the more potent antifungal activity of EFN, with a narrow range of minimum inhibitory concentration (MIC) values against *T. rubrum* (0.001 − 0.015 μg/mL) and *T. mentagrophytes* (0.001 − 0.03 μg/mL) compared to those of ciclopirox (0.03 − 0.5 μg/mL for *T. rubrum* and 0.03 − 0.5 for *T. mentagrophytes*), as well as rapid and enhanced drug absorption from the topical solutions compared to that from nail lacquers (Jo Siu et al., 2013; Sugiura et al., 2014).

Although EFN-K exhibited more rapid and enhanced penetration though the skin and nail plate than reference control #2 and EFN-J, it showed similar antifungal efficacy to reference control #1 and EFN-K; moreover, greater structural recovery of the keratin layer was seen with EFN-K. This may have been due to the sustained action of the accumulated EFN in the nail plate with repeated administration of the EFN topical solutions over a long period. Further *in vivo* studies are required to compare the time to recovery and complete cure rates of EFN-K to the reference controls, to fully understand the improved skin and nail infiltration properties.

Taken together, the results presented herein imply that the enhanced absorption of EFN-K should improve patient compliance, such that it can serve as a rapid local treatment for onychomycosis.

## 4. Conclusions

In this study, 10% EFN topical solutions were prepared to improve the transungual permeability and antifungal efficacy of EFN through the introduction of Transcutol P and isopropyl myristate as permeation enhancers, and cyclomethicone as a wetting agent. In addition, the optimum EFN-K, which was stabilized by adding BHT, DTPA, and citric acid as antioxidant, chelating, and pH-adjusting agents, respectively, exhibited 1.18-fold higher *in vitro* antifungal activity against *T. rubrum* than the reference control #1. EFN-K showed a significant (1.46-fold) increase in the penetration of EFN across full-thickness human skin compared to reference control #1 (commercial 10% EFN topical solution) after a 24-hour incubation. Furthermore, after the 24-hour incubation EFN-K showed 1.46- and 1.33-fold greater human nail permeation and infiltration into the nail plate than the reference control #1, respectively, which in turn significantly increased the nail flux on day 7 (by 89.7%) compared to the reference control #1. As a result, EFN-K markedly decreased the mean viable fungal cell count (by 54.3%) compared to the vehicle-treated group after once-daily treatment for 4 weeks in the *T. mentagrophytes*-infected guinea pig onychomycosis model. These observations suggest that highly skin- and nail-permeable EFN-K can improve patient compliance and increase the cure rate of onychomycosis.

## Supplementary Material

Supplemental Material
