# Supplementary material for: Preparation and in vivo evaluation of a highly skin- and nail-permeable efinaconazole topical formulation for enhanced treatment of onychomycosis
Source: Drug Deliv. 2019 Nov 18;26(1):1167–77. doi: 10.1080/10717544.2019.1687612 (PMC6882438; doi:10.1080/10717544.2019.1687612)
Supplement: Supplemental Material [file IDRD_A_1687612_SM9852.docx]

**Supporting information**

**Preparation and *in vivo* evaluation of a highly skin- and nail-permeable efinaconazole topical formulation for enhanced treatment of onychomycosis**

Byung Chul Lee^a,b^, Pangeni Rudra^c^, Jungtae Na^a,b^, Kyo-tan Koo^d^, Jin Woo Park^c^

*^a^* Department of Dermatology, College of Medicine, Chung-Ang University, Seoul 06974, Republic of Korea

*^b^* Department of Medicine, Graduate school, Chung-Ang University, Seoul 06974, Republic of Korea

*^c^* College of Pharmacy and Natural Medicine Research Institute, Mokpo National University, Jeonnam 58554, Republic of Korea

*^d^* BioBelief, Seoul 05841, Republic of Korea

**Supplemental data**

**(A) (B)**

**(C)**

**Figure S1.** Discoloration of the reference control #1 (A), EFN-J (B), and EFN-K (C) solutions was evaluated by measuring absorbance at 400, 500, and 600 nm after storage at 65 °C for 5 weeks. Each value represents the mean ± standard deviation (*n* = 3).
